# An unusual presentation of an intraosseous epidermoid cyst of the anterior maxilla: a case report

**DOI:** 10.1186/1752-1947-8-262

**Published:** 2014-07-28

**Authors:** Sinan Y Ertem, Sina Uckan, Handan Ozdemir

**Affiliations:** 1 Department of Oral and Maxillofacial Surgery, School of Dentistry, Kırıkkale University, Kırıkkale, Turkey; 2 Department of Oral and Maxillofacial Surgery, School of Dentistry, Medipol University, İstanbul, Turkey; 3 Head of Department of Pathology, School of Medicine, Baskent University, Ankara, Turkey; 4 Kırıkkale Universitesi, Dis Hekimligi Fakultesi, Kurtulus mah. 692. Sok no: 31 Merkez, Kırıkkale, Turkey

**Keywords:** Cyst, Epidermoid, Intraosseous

## Abstract

**Introduction:**

Intraosseous epidermoid inclusion cysts are rare benign epithelial inclusion cysts in the bone. They are usually found in the cranium and hand phalanges. They are slow growing lesions, and it is difficult to differentiate them from other inflammatory and cystic lesions. Only a few cases of epidermoid inclusion cyst in the jaw have been reported in the literature. This is the fourth case reported as intraosseous epidermoid cyst of the maxilla in the English literature.

**Case presentation:**

An asymptomatic 59-year-old Caucasian man was referred to our Oral and Maxillofacial Surgery clinic for a unilocular radiolucent area at his anterior maxilla shown on an orthopantomograph. He was scheduled for surgery and underwent cyst extraction surgery. A pathological examination revealed epidermoid cyst. The diagnostic dilemma in this case report in opposition to the presented intraosseous epidermoid cysts in the literature is that there was no trauma history to his upper jaw. Treatment for this cyst is conservative surgical excision and recurrence is uncommon.

**Conclusions:**

This report presents an unusual case of an intraosseous epidermoid cyst that occurred with no trauma history to the upper jaw. Although only three cases of epidermoid inclusion cyst have been reported in the maxilla, epidermoid inclusion cyst should be considered in the differential diagnosis of radiolucent lesions of the jaws.

## Introduction

An epidermoid cyst is a benign soft tissue neoplasm that can be seen anywhere in the body. Males are affected more frequently than females. This lesion is unusual before puberty unless associated with Gardner syndrome [1]. Epidermoid cysts are nodular, fluctuant, subcutaneous lesions and are confined to one anatomical area. There may be relation with inflammation or not. These cysts are soft tissue tumors and are rarely seen in the head and neck region (7%). Only 1.6% are located within the oral cavity [2,3]. The general intraoral localization of this cyst is the floor of the mouth, the rest are found in the tongue, lips, palate, jaws and cheek [4]. If established at the mouth floor, it will uphold the tongue and may prevent speech and chewing. The clinical presentation of this lesion is localized and the consistency of the cyst is doughy and fluid.

## Case presentation

**Figure 1:**
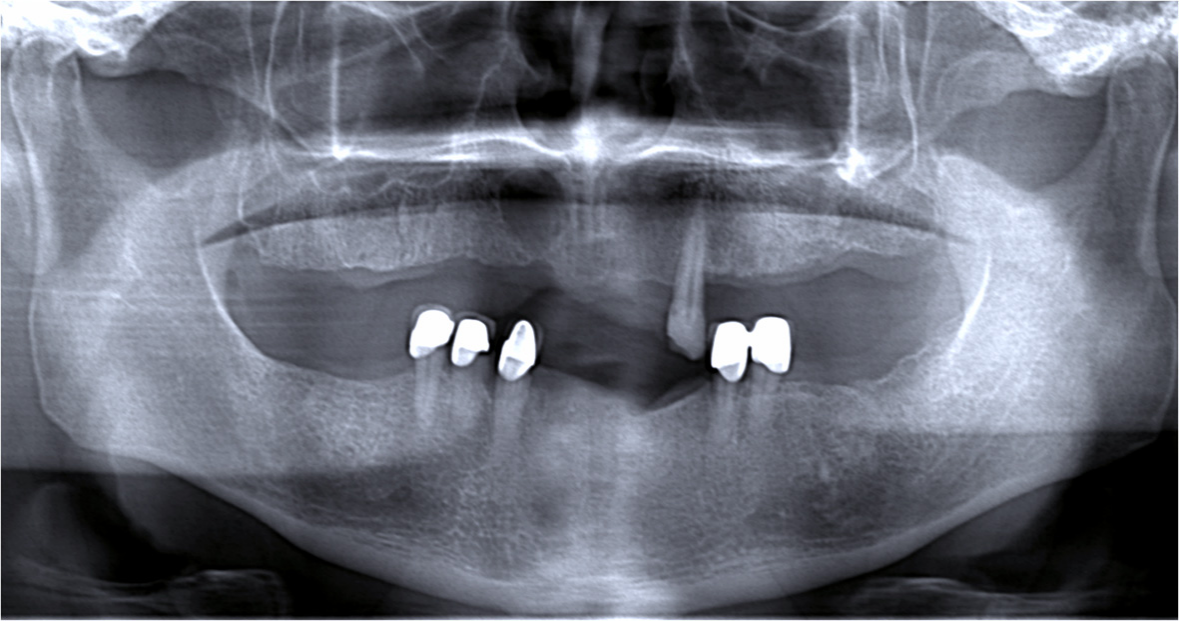
Orthopantomograph showing unilocular radiolucency.

**Figure 2:**
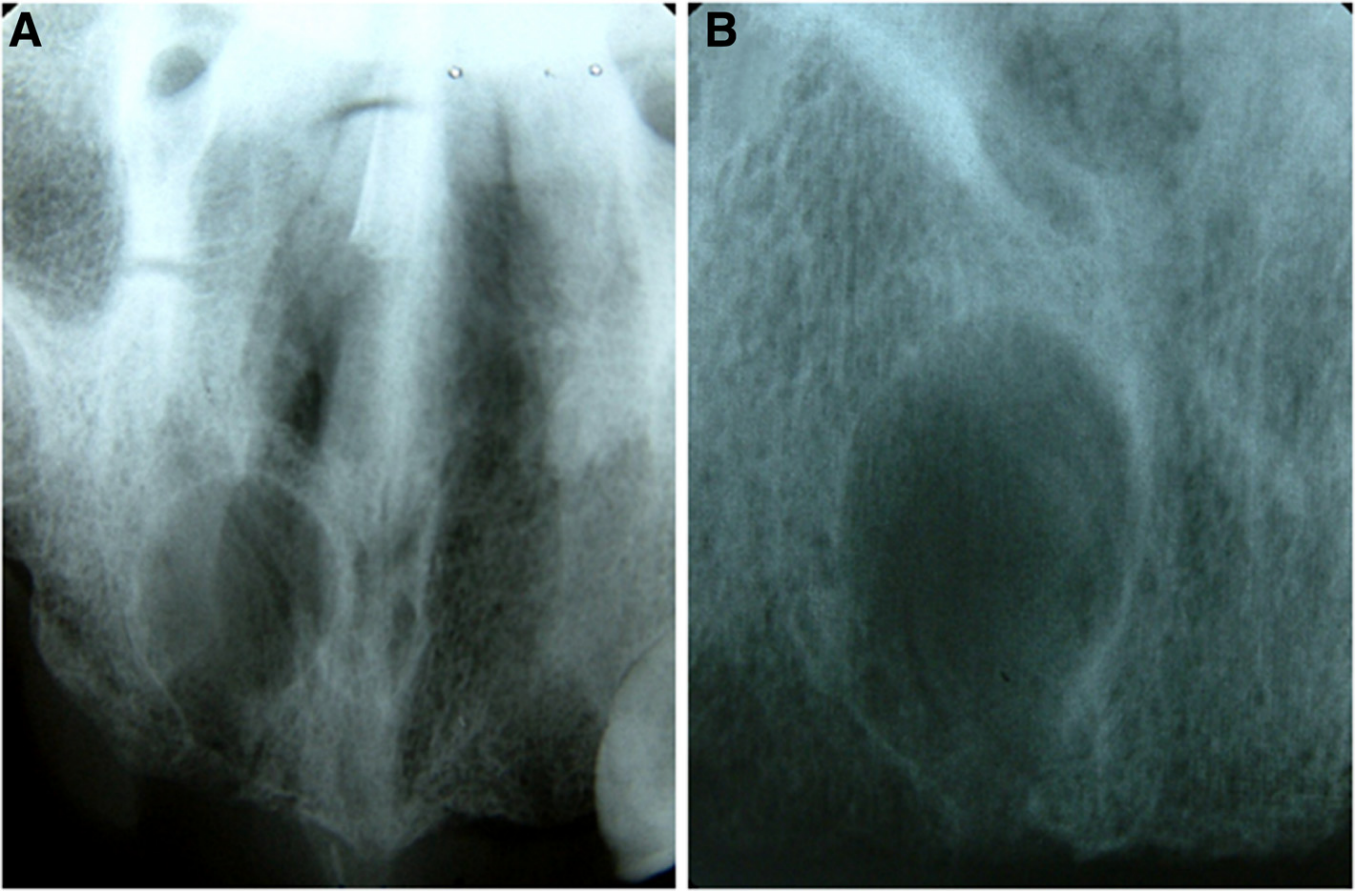
**Occlusal and periapical radiographs showing intraosseous radiolucent area with sclerotic border. A**. Occlusal radiograph **B**. Periapical radiograph.

An asymptomatic 59-year-old Caucasian man was referred to the clinic for routine dental evaluation. A radiologic examination revealed a unilocular radiolucent area at his anterior right maxilla with well-circumscribed sclerotic border (Figures 1 and 2).

All his maxillary teeth with the exception of his left maxillary cuspid had been removed over the years, uneventfully; the most recent extractions were performed 7 years previously. There was no history of trauma to his maxilla or of any other surgery. He was a non-cigarette smoker, had no further illness and was otherwise healthy. On examination, the alveolus was of normal consistency with expansion or swelling; there was no pain or tenderness; there was no change in the color of the soft tissues around the lesion. There was no lymphadenopathy.

Based on the clinical and radiographic examinations, differential diagnosis should be performed with other radiolucent lesions such as residual, radicular or lateral periodontal cyst, odontogenic keratocyst, globulomaxillary cyst, traumatic bone cyst and ameloblastoma.

Residual cyst is the first pathology that should be considered depending on the similarity of radiologic appearance, clinical evaluation and location, unless the lesion is located around the tooth root region and the lesion is unilocular and asymptomatic as in the presented case.

On the one hand residual lateral periodontal cysts occur along remnants of the lateral root surface of a tooth and 80% occur in the mandibular premolar, canine, lateral incisor area. On the other hand, polycystic appearance may be seen radiologically.

A unilocular clinical appearance can also be seen with odontogenic keratocysts. The usual localization of keratocysts is the posterior mandible and ascending ramus and for large keratocysts their radiologic appearance can be multilocular. In addition, in up to 40% of odontogenic keratocysts an unerupted tooth is involved in the lesion.

Globulomaxillary cysts are one of the pathologic lesions seen in this region of the maxilla. On clinical examination this cyst is localized between the maxillary lateral and canine tooth; radiological evaluation shows unilocular radiolucency. Although recently most so-called globulomaxillary cysts are usually found to be odontogenic cysts of various types, it should be also considered for differential diagnosis.

Traumatic bone cyst should be discussed in the diagnosis. A generally agreed etiologic factor for this cyst is bleeding into the bone after trauma. Organized hematoma will develop the cystic defect. Similarly, according to the literature, the most probable etiologic factor for intraosseous epidermoid cysts is traumatic implantation of epidermoid cells into the bone. This lesion is usually seen at the first and second decades of life primarily at the premolar and molar area of the mandible. The lesion is usually asymptomatic but sometimes pain or paresthesia occurs. The radiologic appearance is well-defined unilocular radiolucency.

**Figure 3:**
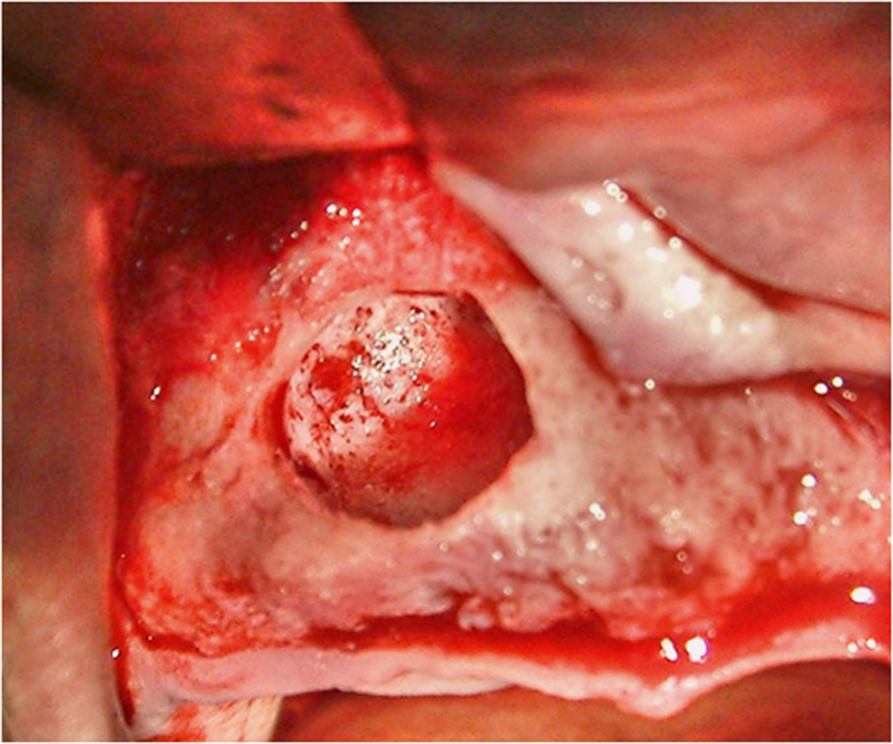
Cavity after cyst extracted.

Ameloblastomas generally occur at the posterior mandible and are mostly seen in multicystic (86%) form. Unilocular lesions are mostly localized at the pericoronal area of an unerupted mandibular third molar.The patient was scheduled for surgery. Under aseptic conditions, his anterior superior alveolar nerve was blocked with local infiltration using 4% articaine hydrochloride and 1:100,000 epinephrine. A full thickness mucoperiosteal envelope flap with two oblique release incisions was carried out to expose the lesion and the surrounding alveolar bone was removed by round and fissure burs under irrigation with sterile saline solution. On clinical examination the lesion appeared well demarcated from the surrounding bone. The unilocular mass was removed totally (Figure 3). Sharp edges were rounded and the wound was closed with 3–0 vicryl suture. It was noted that the pathologic lesion was surrounded by resistant lining with fluid inside. The wound healed satisfactorily, and there was no recurrence during the follow-up period of 36 months.

**Figure 4:**
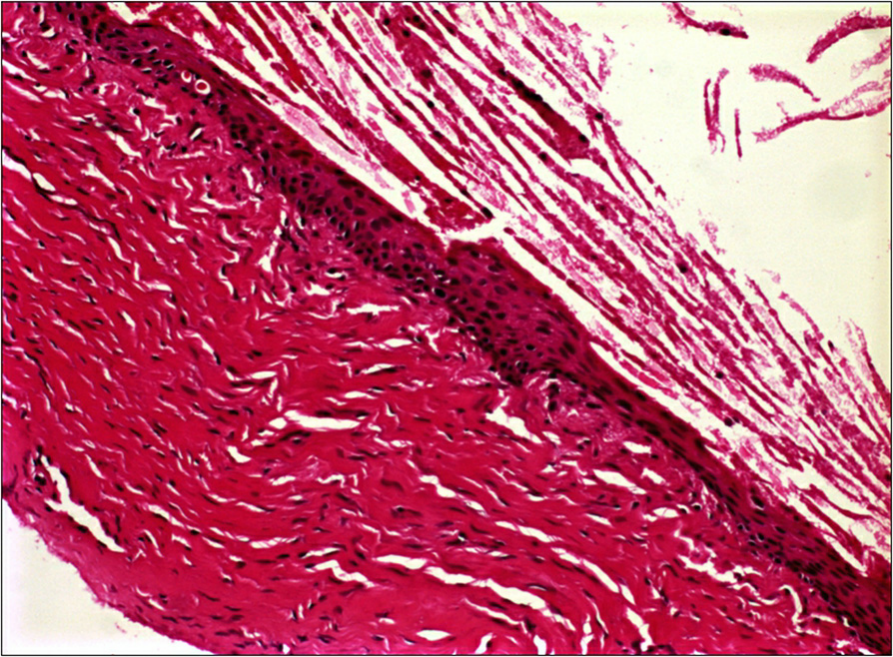
**Note the cyst-filled space containing laminated keratin.** The cyst is lined by stratified squamous epithelium. Hematoxylin and eosin stain, original magnification, 200×.

The gross pathologic specimen was a well-circumscribed cystic lesion containing soft, tannish yellow material in cross-section. Grossly the extracted cystic lesion measured 1.0×1.0×0.7cm.Microscopic examination of the specimen revealed a cavity that was lined with stratified squamous epithelium resembling epidermis (Figure 4). A well-developed granular cell layer was seen and the lumen was filled with regenerating orthokeratin. The final diagnosis was epidermoid cyst.

## Discussion

The usual etiologic factor for epidermoid cyst generation is localized inflammation of hair follicles. Other etiologic factors are non-neoplastic proliferation of infundibular epithelium during healing process or traumatic implantation of dermis epithelium [5-7].

Histological differential diagnosis should eliminate dermoid cysts. To be designated as a dermoid cyst, skin appendages, such as hair follicles, sebaceous and sweat glands, and arrector pili muscles must be identified in the wall of the cyst. As the specimen presented here did not contain these dermal tissues it was defined as an epidermoid cyst.

The development of epidermoid cysts can be due to congenital or acquired factors. The former is thought to develop from congenital inclusion of ectodermal tissue during embryological development [4]. Although the generally agreed etiologic factor is congenital, trauma was reported as the possible cause of this lesion. Clinicians claim that traumatic implantation of cystic cells into deeper tissues with subsequent cystic change and expansion is the major etiological factor for formation of intraosseous epidermoid cysts [5-7]. This lesion also can originate from hard tissues such as calvarium, temporal bone and sphenoid bone [2]. Dammert *et al*. reported that head injuries caused the inclusion of epidermal cells into the calvarium, and epidermoid cysts occurred [8].

Craig *et al*. presented the first fully documented example of a totally intraosseous dermoid cyst of the mandible in 1980 [9]. Following this article six more intraosseous dermoid cyst cases were presented. Reports of epidermoid cysts at jaw bones are even lower than dermoid cysts. A few cases of maxillary intraosseous epidermoid cysts have been reported in the English literature [2,10]. The prognosis of epidermoid cyst is fairly good and treatment consists of conservative surgical excision.

## Conclusion

Although intraosseous epidermoid cysts are extremely rare in the maxilla, they should be considered in the differential diagnosis of radiolucent lesions of the jaws.

## Consent

Written informed consent was obtained from the patient for publication of this case report and accompanying images. A copy of the written consent is available for review by the Editor-in-Chief of this journal.

## Competing interests

The authors declare that they have no competing interests.

## Authors’ contributions

SYE was the principal author and major contributor in writing the manuscript. SU collaborated in writing the manuscript. HO performed the histological examination of the epidermoid cyst. All authors reviewed and approved the final manuscript.
